# Case report of a novel homozygous splice site mutation in *PLA2G6* gene causing infantile neuroaxonal dystrophy in a Sudanese family

**DOI:** 10.1186/s12881-018-0592-y

**Published:** 2018-05-08

**Authors:** Liena E. O. Elsayed, Inaam N. Mohammed, Ahlam A. A. Hamed, Maha A. Elseed, Mustafa A. M. Salih, Ashraf Yahia, Rayan A. Siddig, Mutaz Amin, Mahmoud Koko, Mustafa I. Elbashir, Muntaser E. Ibrahim, Alexis Brice, Ammar E. Ahmed, Giovanni Stevanin

**Affiliations:** 1 Faculty of Medicine, University of Khartoum, Qasr Street, 11111 Khartoum, Sudan; 2 Division of Pediatric Neurology, Department of Pediatrics, College of Medicine, King Saud University, Riyadh, Saudi Arabia; 3 Department of Molecular Biology, Institute of Endemic Diseases, University of Khartoum, Khartoum, Sudan; 4 Institut du Cerveau et de la Moelle épinière, INSERM U1127, CNRS UMR7225, Sorbonne Universités, UPMC Université Paris VI UMR_S1127, 75013 Paris, France; 5 Ecole Pratique des Hautes Etudes, EPHE, PSL research university, 75014 Paris, France; 6 Department of genetics, APHP Pitié-Salpêtrière Hospital, 75013 Paris, France; 7 Department of Biochemistry, Faculty of Medicine, National University, Khartoum, Sudan; 8 Department of Neurology and Epileptology, Hertie Institute for Clinical Brain Research, Tuebingen, Germany

**Keywords:** Infantile neuroaxonal dystrophy, PLA2G6, Whole exome sequencing, Sudan

## Abstract

**Background:**

Infantile neuroaxonal dystrophy (INAD) is a rare hereditary neurological disorder caused by mutations in *PLA2G6*. The disease commonly affects children below 3 years of age and presents with delay in motor skills, optic atrophy and progressive spastic tetraparesis. Studies of INAD in Africa are extremely rare, and genetic studies from Sub Saharan Africa are almost non-existent.

**Case presentation:**

Two Sudanese siblings presented, at ages 18 and 24 months, with regression in both motor milestones and speech development and hyper-reflexia. Brain MRI showed bilateral and symmetrical T2/FLAIR hyperintense signal changes in periventricular areas and basal ganglia and mild cerebellar atrophy. Whole exome sequencing with confirmatory Sanger sequencing were performed for the two patients and healthy family members. A novel variant (NM_003560.2 c.1427 + 2 T > C) acting on a splice donor site and predicted to lead to skipping of exon 10 was found in *PLA2G6*. It was found in a homozygous state in the two patients and homozygous reference or heterozygous in five healthy family members.

**Conclusion:**

This variant has one very strong (loss of function mutation) and three supporting evidences for its pathogenicity (segregation with the disease, multiple computational evidence and specific patients’ phenotype). Therefore this variant can be currently annotated as “pathogenic”. This is the first study to report mutations in *PLA2G6* gene in patients from Sudan.

## Background

Infantile neuroaxonal dystrophy (INAD) is a rare hereditary neurological disorder caused by mutations in the *PLA2G6* gene [1]. The disease commonly affects children below 3 years of age and presents with delay in motor skills, optic atrophy and progressive spastic tetraparesis. Brain MRI shows cerebellar atrophy, white matter abnormalities and hypointense globus pallidus. Infants with the disease are usually normal at birth but deteriorate rapidly and rarely live beyond their first decade [2].

The incidence and prevalence of INAD is currently unknown [3]. More than 85% of cases are caused by mutations in *PLA2G6* gene, while the remainder has unknown genetic cause. The protein product of *PLA2G6* gene has important roles in the metabolism of membrane phospholipids. Sequencing analysis of *PLA2G6* gene has identified missense, nonsense, splicing disruption variants and insertion/deletions with genotype/phenotype correlation: loss of function mutations are usually associated with more severe forms of the disease, while compound heterozygous variants have usually milder presentation [4].

Studies of INAD in Africa are extremely rare, and genetic studies from Sub Saharan Africa are almost non-existent. In this first study of INAD from Sudan, we are reporting a novel homozygous splice site mutation in *PLA2G6* gene in two siblings with INAD.

## Case presentation

**Fig. 1:**
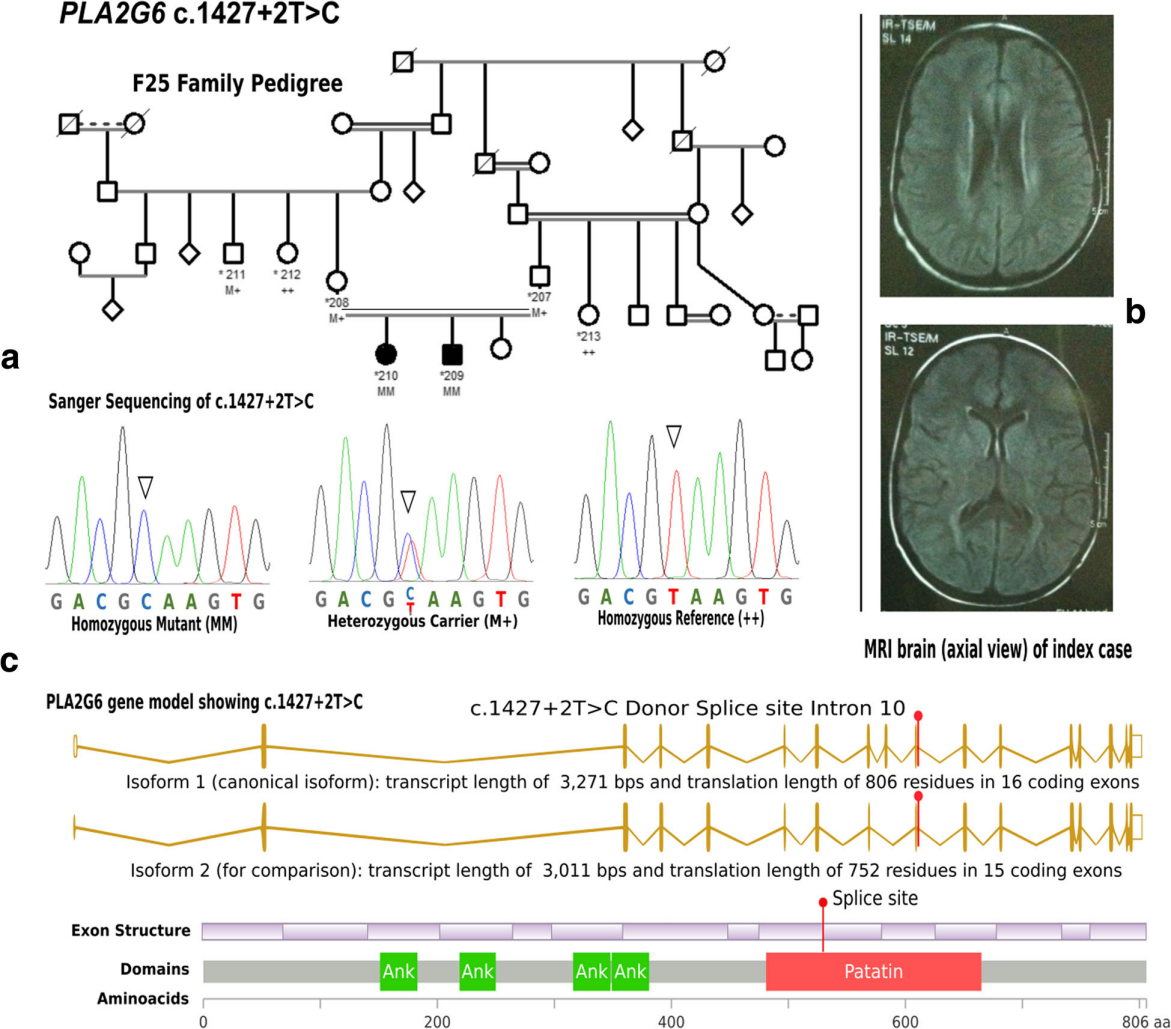
**a** Pedigree and MRI of the index patient (210) of family F25 caused by splice donor mutation in *PLA2G6* segregating with the disease distribution in whole family presenting with pyramidal signs and features associated with infantile neuroaxonal dystrophy (INAD). **b** MRI shows bilateral and symmetrical T2/FLAIR hyperintense signal changes in periventricular areas, basal ganglia, globus pallidus and to lesser degree head of caudate nucleus and putamen. **c** Chromotagram of Sanger sequencing showing homozygous mutation in proband, a heterozygous carrier and a control homozygous reference allele with conserved amino acid sequence. Pedigree symbols: * sampled individual; Phenotype symbols: black color: affected individuals; Genotype symbols: ++ Homozygous reference genotype; M+ Heterozygous genotype; MM Homozygous mutant genotype. Others are standard medical pedigree symbols

Two Sudanese siblings from a consanguineous family (F25) from White Nile area in central Sudan presented with delayed motor and intellectual development. Patient 1 presented at age 24 months with walking difficulty due to muscle weakness and poor speech. The patient was able to sit without support at age 6 months; she started walking with assistance at age 12 months but lost her ability to walk at age 2 years. She was able to say “dada, mama” at age 8 months but lost this ability by age 20 months. At age 2.5 years, the patient became non-ambulatory and completely lost her speech ability. Patient 2 presented with the same clinical features but started earlier at age 18 months, he was able to sit with support and say “dada, mama” at ages 6 and 8 months respectively. He was able to walk with support at age 12 months but at the time of examination, he was only able to roll in bed. Both patients had progressive dysphagia for both solids and liquids and behavioral changes (lack of communication in patient 1 and significant irritability in patient 2). On examination both patients had normal upper and lower limbs tone and hyperreflexia in all limbs with up-going plantar response. Cognitive status of the two patients was difficult assess but both patients appeared to be non-attentive. Both patients had marked left sided visual impairment and bilateral optic atrophy. There were no extrapyramidal signs. Brain MRI showed bilateral and symmetrical T2/FLAIR hyperintense signal changes in periventricular areas, basal ganglia, globus pallidus and to lesser degree head of caudate nucleus and putamen as well as the cerebellum. Mild generalized cerebellar atrophy was found in both patients but there was no evidence of brain iron accumulation (hypointense signals in T2 and hyperintense signals in T1 weighted images), Fig. 1.

### Whole exome sequencing (WES)

Two milliliters of saliva were collected using Oragene.Discover DNA collection kits (DNA Genotek Inc., Ottawa, ON, Canada). DNA was extracted according to prepIT.L2P manual protocol. Standard Agarose gel electrophoresis, NanoDrop spectrophotometer (Thermo Scientific, Wilmington, DE, USA) and Qubit fluorometer (Promega, Madison, WI, USA) were used to qualify and quantify DNA. Sequencing was performed using the MiSeq platform (Illumina, San Diego, CA, USA) on 250 bp paired-end reads according to the manufacturer’s recommendations. Variant calling and quality control were performed using Genomics Workbench (CLC Bio, Aarhus, Denmark). SNVs and Indels were detected using probability-based and quality-based algorithms. For gene/variant prioritization, nonsense, frameshift, splice site and missense variants with a minimum depth of 30 × were selected. Minor allele frequency cutoff of 1% was used.

### Sanger sequencing

To validate whole exome sequence results and segregation analysis we performed Sanger sequencing for the candidate variants for the two patients and five healthy family members using the BIGDYE chemistry on an ABI3730 sequencer (Applied Biosystems). Seqscape (Applied Biosystems) and Chromas lite software (Technelysium, South Brisbane, QLD, Australia) were used for sequence analysis and visualization. Genetic testing was done for the two patients and their parents and all available family members at the time of presentation.

### Results

WES analysis identified one novel variant (NM_003560.2 c.1427 + 2 T > C) in the *PLA2G6* gene as affecting a splice site at the exon 10-intron 10 junction. The mutation is predicted to disrupt splicing and skipping of exon10 with high (100%) confidence score using three different splice prediction tools: MaxEnt (http://genes.mit.edu/burgelab/maxent/Xmaxentscan_scoreseq.html), NNSPLICE (http://www.fruitfly.org/seq_tools/splice.html) and HSF (http://www.umd.be/HSF3/). The mutation co-segregated with the disease in the family. It was found in a homozygous state in the two patients and homozygous reference or heterozygous in five healthy related individuals (Fig. 1).

## Discussion and conclusion

INAD is a typical example of a rare hereditary neurological disease with high mortality and morbidity [2]. Mutations in *PLA2G6* gene causing INAD have been described previously in many studies [4–13]. These were more commonly homozygous missense variants or deletions which lead to loss of protein function. Splice site variants have been rarely reported although they are considered also loss of function mutations [14]. The spectrum of causative mutations is obscure and possibly unknown in patients from Sub Saharan Africa despite the well-known genetic heterogeneity in this region [15]. This is the first study of INAD in Sudan and it showed a novel homozygous splicing mutation (c.1427 + 2 T > C) in exon 10 of *PLA2G6* gene of two siblings with typical clinical features. The variant segregated with the disease; both parents were heterozygous and all unaffected family members had either the wild type genotype or were heterozygous. Mutations in exon 10 have been previously reported in association with INAD in dogs [16]. This comports with its role in the pathogenesis of the disease in this family. The effect on mRNA could not be checked however as no cells were available from the patients.

According to the latest ACMG guidelines [17], this variant has one very strong (loss of function mutation) and three supporting evidence for its pathogenicity (segregation with the disease, multiple computational evidence and specific patients’ phenotype). In addition, this variant was absent in Exac and 1000 genome browser databases and has a total allele frequency of 0.4065 × 10^− 5^ in genomAd browser with no reported homozygotes. Furthermore, the (c.1427 + 2 T > C) variant is highly predicted to cause skipping of exon 10 during mRNA maturation. Splicing alteration leads to skipping of an exon or retention of an intron both significantly altering protein structure and function [18]. Therefore this variant can be currently annotated as “pathogenic”.

Mutations in *PLA2G6* gene continue to be discovered from throughout the world, but our study is the first to report cases of INAD with associated genetic changes from Sudan. However functional studies are still needed to verify the effect of the reported mutation on the expression and function of PLA2G6 protein.

## Abbreviations

ACMG: American College of Medical Genetics
DNA: Deoxyribonucleic acid
INAD: Infantile neuroaxonal dystrophy
